# Oligomerization Profile of Human Transthyretin Variants with Distinct Amyloidogenicity

**DOI:** 10.3390/molecules25235698

**Published:** 2020-12-03

**Authors:** Ana Frangolho, Bruno E. Correia, Daniela C. Vaz, Zaida L. Almeida, Rui M. M. Brito

**Affiliations:** 1 Chemistry Department and Coimbra Chemistry Centre, University of Coimbra, 3004-535 Coimbra, Portugal; ana.frangolho@inl.int (A.F.); bruno.correia@epfl.ch (B.E.C.); daniela.vaz@ipleiria.pt (D.C.V.); zalmeida@qui.uc.pt (Z.L.A.); 2 International Iberian Nanotechnology Laboratory, 4715-330 Braga, Portugal; 3 École Polytechnique Fédérale de Lausanne, CH-1015 Lausanne, Switzerland; 4 CiTechCare, Center for Innovative Care and Health Technology, School of Health Sciences, Polytechnic Institute of Leiria, 2411-901 Leiria, Portugal

**Keywords:** transthyretin, TTR, TTR variants, amyloidosis, ATTR, linear oligomerization, downhill polymerization, aggregation, amyloid

## Abstract

One of the molecular hallmarks of amyloidoses is ordered protein aggregation involving the initial formation of soluble protein oligomers that eventually grow into insoluble fibrils. The identification and characterization of molecular species critical for amyloid fibril formation and disease development have been the focus of intense analysis in the literature. Here, using photo-induced cross-linking of unmodified proteins (PICUP), we studied the early stages of oligomerization of human transthyretin (TTR), a plasma protein involved in amyloid diseases (ATTR amyloidosis) with multiple clinical manifestations. Upon comparison, the oligomerization processes of wild-type TTR (TTRwt) and several TTR variants (TTRV30M, TTRL55P, and TTRT119M) clearly show distinct oligomerization kinetics for the amyloidogenic variants but a similar oligomerization mechanism. The oligomerization kinetics of the TTR amyloidogenic variants under analysis showed a good correlation with their amyloidogenic potential, with the most amyloidogenic variants aggregating faster (TTRL55P > TTRV30M > TTRwt). Moreover, the early stage oligomerization mechanism for these variants involves stepwise addition of monomeric units to the growing oligomer. A completely different behavior was observed for the nonamyloidogenic TTRT119M variant, which does not form oligomers in the same acidic conditions and even for longer incubation times. Thorough characterization of the initial steps of TTR oligomerization is critical for better understanding the origin of ATTR cytotoxicity and developing novel therapeutic strategies for the treatment of ATTR amyloidosis.

## 1. Introduction

Transthyretin (TTR) is a homotetrameric protein found in plasma, cerebrospinal fluid, and the eye [1,2,3,4]. Structurally, each TTR monomer is composed of a β-sandwich of two four-stranded β-sheets. TTR’s main known functions are thyroxine hormone (T4) transport and retinol transport in association with retinol-binding protein (RBP) [1,5]. Additionally, TTR is known to have a neuroprotective role in several processes, such as decreasing brain amyloid-β deposition in mouse models [6,7].

TTR is implicated in several acquired and hereditary amyloid pathologies (ATTR, TTR amyloidosis) [1,8,9]. Whereas wild-type TTR (TTRwt) is associated with acquired amyloidosis with mainly cardiac involvement (ATTRwt) [10], different variants of TTR are the cause of hereditary amyloidosis involving the peripheral nervous system, autonomic nervous system, heart, eye, leptomeninges and vasculature of the brain [11,12]. More than 140 amyloidogenic mutations have been described [13], with TTRV30M being the most common amyloidogenic mutation leading to polyneuropathy [14,15], while the TTRL55P variant is associated with one of the most aggressive forms of ATTR polyneuropathy, with an early age of onset at 15–20 years old [16]. TTR amyloidosis shares common characteristics with other amyloid diseases, such as Alzheimer’s, spongiform encephalopathies, or Parkinson’s, where soluble peptides or proteins undergo conformational changes and aggregate into insoluble and highly stable amyloid fibrils [9,17]. Interestingly, the structural and functional dissimilarities between different amyloidogenic proteins lead to amyloid fibrils that share common structural characteristics, namely, the formation of long unbranched filaments with a cross-β-sheet conformation, i.e., β-strands oriented perpendicularly to the fibril main axis [9,17]. Despite the structural resemblance, several distinctive features at the molecular level may explain why the same protein can lead to the manifestation of different pathological phenotypes and amyloid deposition in different organs [18,19].

Previous studies on a wide range of amyloidogenic diseases, including Alzheimer’s, type II diabetes, light-chain amyloidosis (AL), and ATTR, among others, have shown that soluble oligomeric structures and precursors of amyloid fibrils present cytotoxic activity [9,20,21,22,23,24]. Therefore, the accumulation of amyloid deposits is often seen as the last stage of disease pathogenesis, with the intermediate soluble oligomers and aggregates having an important role in triggering cell death. In fact, for TTR, several ex vivo and tissue culture studies have suggested that tissue damage precedes fibril formation and amyloid deposition, indicating that the first steps of amyloid formation trigger pathological processes leading to disease onset [22,25]. Hence, understanding the molecular mechanisms associated with the cascade of events underlying protein oligomerization and amyloidogenesis, in addition to identifying the molecular species involved in protein self-assembly into amyloids, is of paramount importance to develop useful therapeutic strategies to prevent or control amyloid fibril formation [26,27]. 

Previous studies on TTR aggregation showed that amyloid fibril formation is initiated by dissociation of the native tetramer into non-native monomeric species that may aggregate into oligomers prior to amyloid fibril formation [26,27,28,29,30,31]. However, the isolation and characterization of these metastable and polydisperse protein species has been extremely difficult due to the heterogeneous and transient nature of these oligomers. In the present study, we investigated the early stages of TTR oligomerization using photo-induced cross-linking of unmodified proteins (PICUP) [32] and characterized the populations of oligomeric species in solution by sodium dodecyl sulfate polyacrylamide gel electrophoresis (SDS-PAGE), and transmission electron microscopy (TEM). PICUP enables the rapid stabilization of individual oligomers by covalently cross-linking protein molecules, which are closely associated. Thus, PICUP has been used as a method to study oligomerization processes and to evaluate the nature of transient oligomers formed by amyloidogenic proteins, such as amyloid-β peptide [33,34,35,36,37,38,39,40,41,42], α-synuclein [43,44,45,46], or prion protein [47], as well as a way to identify relevant targets in the amyloidosis cascade and new therapeutics [37,45]. Here, we compare the oligomerization process of (1) TTRwt; (2) the amyloidogenic variants TTRV30M and TTRL55P; and (3) the nonamyloidogenic variant TTRT119M [25,48,49,50]. Thioflavin-T (ThT) fluorescence assays were also carried out in order to assess the presence of amyloid oligomers/aggregates.

## 2. Results and Discussion

### 2.1. Applicability of PICUP to Study TTR Oligomerization

PICUP has been previously used by several authors to study the oligomerization processes of various amyloidogenic proteins and protein variants, such as the amyloid-β (Aβ) peptide [33,34,35,36,37,38,39,40,41,42], α-synuclein [43,44,45,46], or prion protein [47]. Interestingly, distinct pathways were found for the initial phases of oligomerization of Aβ40 and Aβ42 [33], where only Aβ42 exhibited the formation of pentamer/hexamer units that associate into large oligomers, including dodecamers and octadecamers [20,34,51]. In these studies, Teplow and collaborators used wild-type TTR (TTRwt) as a reference amyloidogenic protein [51,52]. The authors were able to validate the methodology by making use of different proteins and to demonstrate that PICUP is capable of distinguishing between monomeric and different oligomeric states, obtaining different distributions that varied from monomer and dimer [51] to tetramer [52] in the case of TTRwt. Therefore, to study the oligomerization process and species present in the initial steps of TTR aggregation using PICUP, we carefully carried out several cross-linking experiments with TTRwt but also with its amyloidogenic (TTRV30M and TTRL55P) and nonamyloidogenic (TTRT119M) variants.

TTR aggregation was triggered by acidification at pH 3.6, followed by incubation at 25 °C for several hours, and by PICUP as described in Materials and Methods. This acid-induced amyloidogenesis protocol was used as a way of triggering the aggregation process, which otherwise would take several days or weeks (depending on the TTR variant) to occur at pH 7.4, and would not allow the capture of metastable species involved in the initial stages of oligomerization. Moreover, the morphology of the aggregates and amyloid fibrils formed in vitro by TTR, both at acidic and at physiological pH, as well as in vivo, seems to be very consistent in all cases, as confirmed by transmission electron microscopy (TEM) and atomic force microscopy (AFM) data [53].

**Figure 1:**
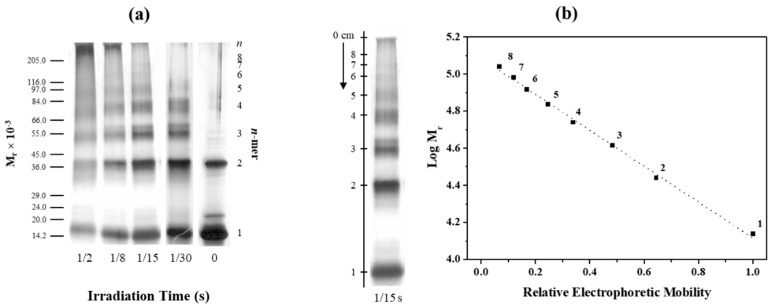
Effect of irradiation time in the PICUP (photo-induced cross-linking of unmodified proteins) experiment on the detection of wild-type transthyretin (TTRwt) oligomer distribution in aggregating samples. (**a**) SDS-PAGE (sodium dodecyl sulfate polyacrylamide gel electrophoresis) of PICUP reaction mixtures performed with irradiation times between 1/30 and 1/2 s. A scale of molecular mass markers is shown on the left. The result for a control experiment, in the absence of light irradiation, is shown on the right lane (0 s). (**b**) Exemplary SDS-PAGE lane (left) and corresponding graph (right) of log(molecular mass) of the (*n* + 1)*mer* (1 to 8) oligomeric species of TTR as a function of the relative electrophoretic mobility. As clearly shown by the linear dependence, the molecular mass of the oligomers is consistent with the sequential addition of approximately 14 kDa monomeric units.

The effect of PICUP irradiation time on the detection of oligomer distribution of TTRwt was examined by SDS-PAGE of the reaction mixtures and densitometric quantification of the gels. Figure 1a shows the results obtained when aggregating samples of TTRwt were cross-linked with irradiation times ranging from 1/30 to 1/2 s. Control samples (non-cross-linked, 0 seconds irradiation) and cross-linked samples (1/30, 1/15, 1/8, and 1/2 s) were analyzed by SDS-PAGE. Non-cross-linked aggregating TTRwt (Figure 1a, right-hand lane, 0 s) displayed essentially two protein bands, corresponding to monomer and dimer, while, in contrast, cross-linked TTRwt presented several individual bands. At the shortest exposure time (1/30 s), monomer, dimer, trimer, tetramer, and pentamer forms were clearly observed. With the increase in light exposure time to 1/15 s, sharp gel bands corresponding to higher molecular mass species (pentamer, hexamer, heptamer, octamer) became also visible. As the exposure time increased above 1/15 s (1/15 s to 1/2 s), a gradual decrease in band intensity and sharpness was observed in parallel with the emergence of bands for very high molecular mass species at the top of the gel. This may indicate the formation of non-specific cross-linking at longer times of exposure. Thus, for the lighting set up used, the irradiation time of 1/15 s was chosen to further study the oligomerization process of TTR and its variants. Moreover, according to the mobility of the molecular markers (Figure 1b), different gel bands were found to correspond to different TTR *n-mers*, starting from monomers (~14 kDa) up to at least octamers (~110 kDa), revealing the presence of a “ladder” of *n* + 1 low-molecular-weight (LMW) protein species. Indeed, by plotting the electrophoretic mobility of the protein species as a function of the logarithm of their molecular weights, the data were found to be consistent with the sequential and linear assembly of 14 kDa monomeric units in the oligomerization process of TTRwt.

### 2.2. Early Oligomerization of the Amyloidogenic Variants TTRV30M and TTRL55P

**Figure 2:**
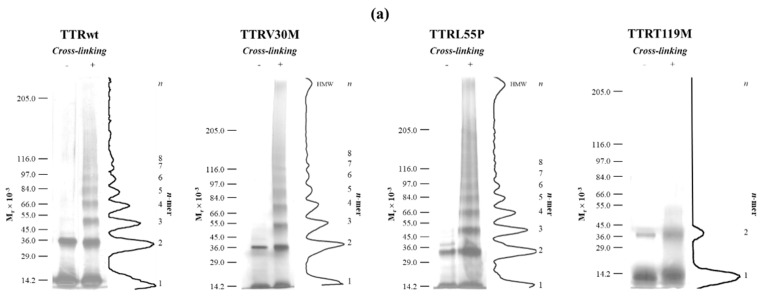

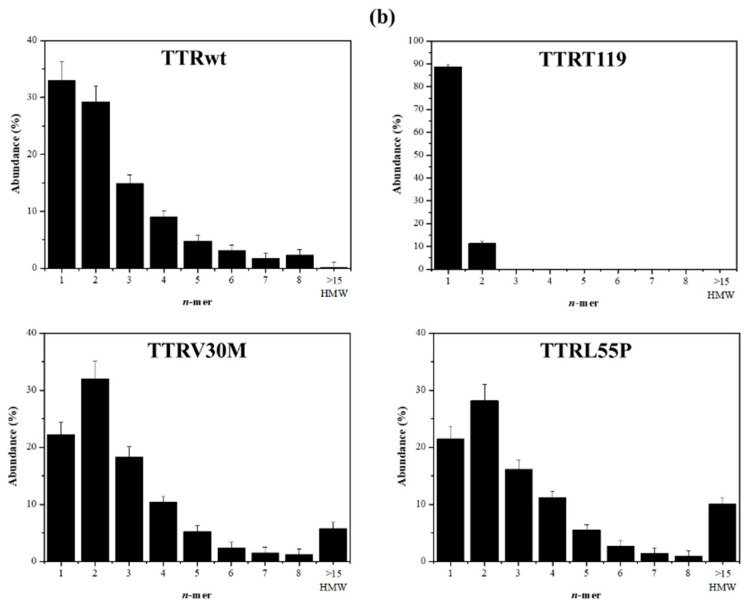
TTR oligomerization followed by photo-induced cross-linking (PICUP) and SDS-PAGE. TTR aggregation was initiated by dilution into acidic conditions (pH 3.6) to a final concentration of 15 μM for TTRV30M, TTRL55P and TTRT119M, and 25 μM for TTRwt, and continued for a period of 15 h at 25 °C. (**a**) Silver-stained SDS-PAGE and densitometric profiles of the oligomerization products of TTR variants (TTRwt, TTRV30M, TTRL55P, and TTRT119M). Non-cross-linked controls are shown in Lane 1 (−). Cross-linked samples are shown in Lane 2 (+). A scale of electrophoretic mobilities of molecular weight markers is shown on the left of each panel and densitometric profiles of Lane 2 on the right. (**b**) Oligomer distributions for different TTR variants plotted as a percentage of the total amount for each lane versus *n-mer* order.

The first steps of the oligomerization process of the naturally occurring amyloidogenic variants TTRV30M and TTRL55P were characterized using the PICUP workflow at pH 3.6 and at pH 7.4. While at pH 7.4 no oligomers were observed at any of the irradiation times tested (Figure S1, Supplementary Materials), at pH 3.6 a ladder-like pattern of protein species was observed (Figure 2). Both cross-linked and non-cross-linked samples were analyzed by SDS-PAGE in the first steps of the oligomerization mechanism of TTR under acidic pH. As for TTRwt, the same *n-mer* pattern was observed (Figure 2a). Both the TTRV30M and TTRL55P variants showed only two major individual gel bands in the non-cross-linked control samples (Figure 2, left-hand lanes) and presented an *n* + 1 oligomeric pattern from 1-*mers* to at least 8-*mers* (~14 to 110 kDa), in the PICUP “ladder” (right-hand lanes).

Moreover, the oligomerization process occurred more rapidly for the V30M and L55P amyloidogenic variants than for TTRwt, since after only 15 h of incubation, high-molecular-weight (HMW) species could be detected for both variants at the top of the gels (Figure 2a, right-hand lanes), in contrast to TTRwt. In addition, the molecular population distributions of TTR oligomers formed by acidic treatment were also determined (Figure 2b) and were found to be distinct depending on the TTR variant under study. The relative abundance of HMW species formed by TTRV30M within less than a day was lower than that for TTRL55P, and this characteristic is related to the amyloidogenic potential of the variants [28,54]. At the early stages of the oligomerization process (15 h of incubation), the SDS-PAGE gels showed that the most abundant species were 1-*mers* (22.2% and 21.5% for TTRV30M and TTRL55P, respectively, compared to 33.0% for TTRwt) and 2-*mers* (32.0% and 28.2% for TTRV30M and TTRL55P, respectively, compared to 29.2% for TTRwt), with TTRL55P showing higher amounts of larger-sized oligomers (>3-*mers*) and HMW species (Figure 2). Nonetheless, regardless of the protein variant and oligomerization kinetics, the same pattern of linear polymerization was observed for the three TTR variants, suggesting a downhill polymerization type of oligomerization mechanism through multiple sequential steps, with successive addition of monomeric subunits (1-*mers*) to the growing low-molecular-weight (LMW) oligomer [55], at least until the formation of octamers (8-*mers*) [9]. Additionally, in order to characterize the nature and morphology of the TTR aggregates formed, we have analyzed the samples by thioflavin-T (ThT) extrinsic fluorescence and transmission electron microscopy (TEM).

**Figure 3:**
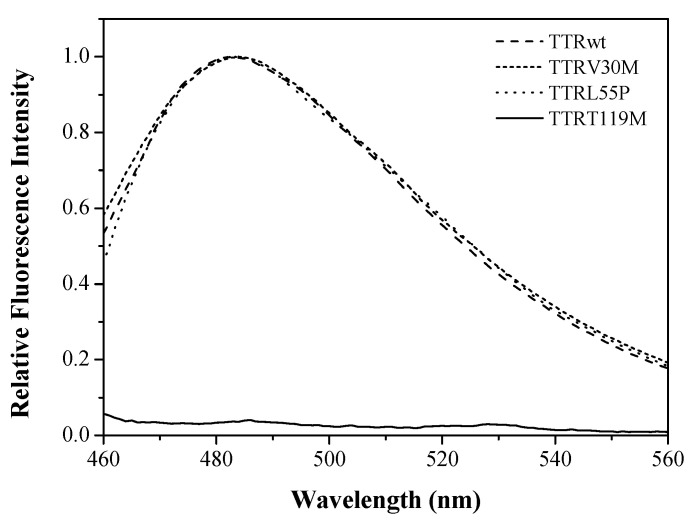
Fluorescence emission spectra of thioflavin-T (ThT) binding to TTR amyloid oligomers of TTRwt, TTRV30M, and TTRL55P. Fluorescence spectra were recorded with an excitation wavelength of 450 nm. Concentrated, fresh, and filtered stock solutions of ThT were prepared in 5 mM glycine–NaOH buffer, pH 9.0. The ThT concentration in the final mixture was 10 μM. In the presence of TTRwt, TTRV30M, and TTRL55P amyloid oligomers, ThT spectra show an increase in florescence intensity and a red shift of the emission maximum to higher wavelengths, whereas in the presence of the nonamyloidogenic variant TTRT119M, no changes were observed.

Amyloid aggregates and fibrils are also known to exhibit distinct tinctorial properties upon binding to Congo red or ThT, among other dyes [9,56,57]. Thus, in order to attest to the amyloid nature of the protein oligomers/aggregates formed by TTR samples upon incubation for 15 h at low pH, ThT fluorescence experiments were carried out (Figure 3). 

**Figure 4:**
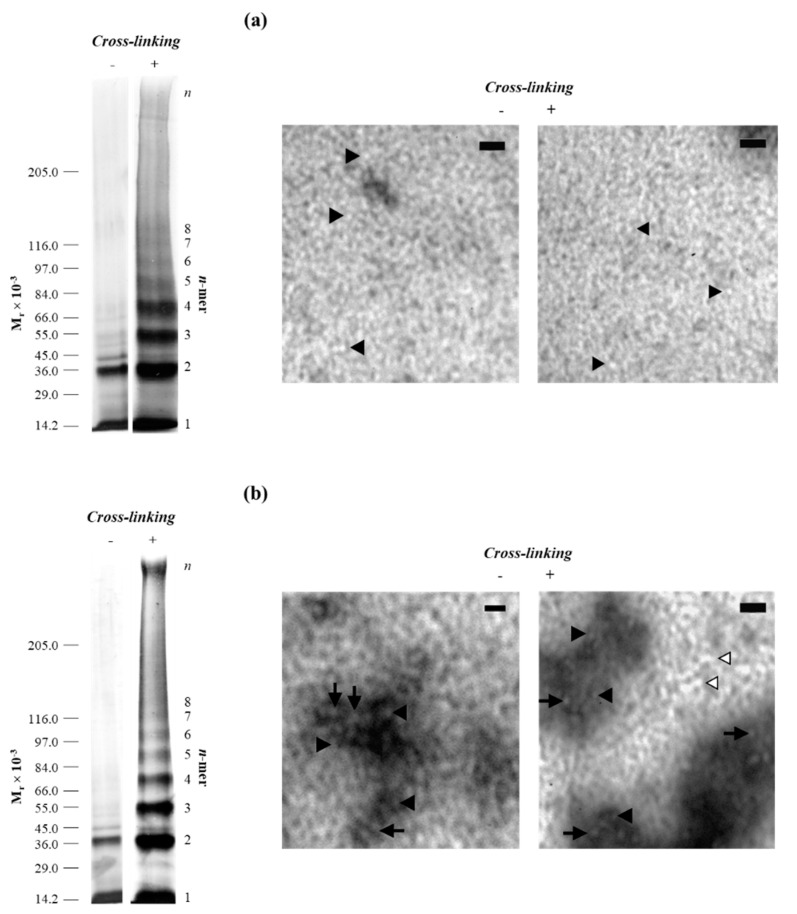
Oligomerization of TTRL55P followed by PICUP and TEM (transmission electron microscopy). TTR samples at 15 μM were incubated for 8 h (**a**) and 72 h (**b**), at pH 3.6, 25 °C. A scale of molecular mass markers is shown on the left of the gels. Non-cross-linked controls (−) and cross-linked samples (+) were analyzed. In the TEM images, black triangles indicate low-molecular-weight (LMW) species that vary from 16 to 23 nm in diameter, while black arrows point towards high-molecular-weight (HMW) species (33–40 nm in diameter), and white triangles indicate unbranched fibrillar structures, with diameters of 33–40 nm, but variable length. Scale bars represent 100 nm.

ThT assays showed an increase in fluorescence intensity of several orders of magnitude along with a red shift of the emission maxima from 445 to 482 nm, confirming the presence of TTR oligomers/aggregates in solution and corroborating the assertion that the soluble aggregates exhibit amyloid characteristics (Figure 3) [9,57]. Moreover, when the aggregates of the most amyloidogenic variant TTRL55P were analyzed by TEM, the amyloid character of the oligomers formed was confirmed. Figure 4 presents TEM images of TTRL55P aggregates, in the absence (−) and presence (+) of PICUP cross-linking, after 8 h (a) and 72 h (b) of incubation at pH 3.6. The cross-linking has neither interfered with the aggregation process nor with the morphology of the TTR aggregates/oligomers. After an 8-h period of incubation (Figure 4a), LMW species (up to octamers) can be found with a diameter of 16–23 nm, as detected by SDS-PAGE and TEM, both in the absence and presence of cross-linking. Likewise, Pires et al. [27] detected the presence of annular oligomers that double-stack into octameric rings with ~16 nm diameter (8 monomers = singlet annulus, and 16 monomers = complete annular doublet). Furthermore, this octameric arrangement may represent a cytotoxic form of the protein, as proposed by Reixach et al. [23]. In addition, after longer incubation periods (72 h) (Figure 4b), larger protein entities were also identified (by SDS-PAGE and TEM), indicating the presence of HMW aggregates and protofibrils, that vary in length. These data confirm the amyloid morphology of the soluble oligomers formed and corroborates the Th-T fluorescence data (Figure 3). In addition, the presence of the cross-linking compounds did not interfere with the structural features of the aggregates obtained, since the structures found were identical in the absence and presence of PICUP.

These results not only corroborate the findings of our previous studies under physiological conditions [28,29,30] but also do agree with previous work on the aggregation mechanism of acidified TTRwt samples where, via SEC-MALS (size exclusion chromatography–multiangle light scattering) and TEM (transmission electron microscopy), it was possible to identify small spherical/annular aggregates (13–16 nm in diameter) composed of 6–10 monomers in the first hours of oligomerization [26]. In addition, octameric annular oligomers ~16 nm in diameter were also found while monitoring the aggregation process of TTRwt upon acidification to pH 3.6 using AFM (atomic force microscopy) [27]. Our data also fits well with the multistep process involving tetramers, intermediates, and higher-order aggregates proposed by Sun et al. [58]. Firstly, TTR tetramers dissociate into monomers, that undergo partial unfolding (and show an aggregation-prone monomeric conformation with different conformational stabilities, depending on the variant) and aggregate to HMW species with amyloid character. In the case of the amyloidogenic variants (TTRV30M and TTRL55P) the kinetics of dissociation, unfolding and aggregation are faster than for TTRwt, a fact that justifies the early disappearance of monomers (which according to Sun et al. are “NMR-invisible”) that is concomitant with an earlier appearance of HMW aggregates (Figure 2) on the pathway to fibrils. Thus, given the metastable nature of these monomeric species, that are difficult to isolate, the present contribution takes advantage of an acid-induced protocol for amyloid formation coupled with PICUP in order to be able to capture the intermediate species involved in the initial steps of the aggregation process that would not be easily isolated and characterized by other methods.

Interestingly, in PICUP studies of oligomerization processes for other amyloidogenic proteins such as the amyloid-β peptide [33,34,35,36,37,38,39,40,41,42], α-synuclein [43,44,45,46], and prion protein [47], the LMW species were found to co-exist in equilibrium with species of higher molecular weight at early stages of the amyloid fibril formation process. As reported for the Aβ40 peptide [51,52], the frequency distributions observed for TTRwt, TTRV30M, and TTRL55P are more consistent with an irregular shape in the low-order oligomer region (monomer to tetramer) and a steep exponential decrease in the abundances of oligomers of an order above tetramers, ending at octamers (Figure 2b). As performed with other amyloid proteins, the PICUP methodology presented here can also be applied to analyze the effect of certain antioxidants (e.g., EGCG and other flavonoids) in the on- and off-pathway aggregation mechanisms for amyloid formation, as a way of inhibiting TTR aggregation and/or promoting the formation of non-toxic conformations [59,60,61,62,63,64].

### 2.3. The Case of the Nonamyloidogenic Variant TTRT119M

**Figure 5:**
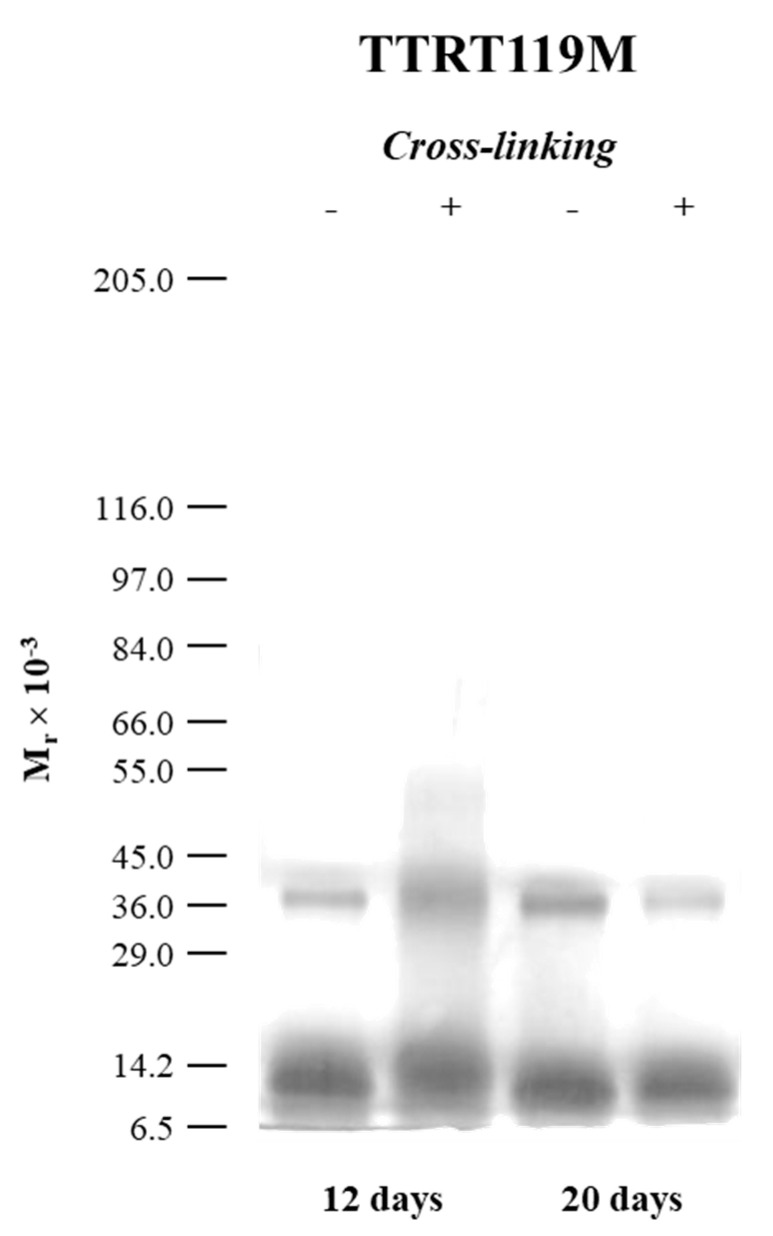
The silver-stained SDS-PAGE profile of TTRT119M after different incubation periods at pH 3.6 and 25 °C, followed by PICUP. Non-cross-linked controls are shown in Lane 1 (−).

The naturally occurring TTRT119M variant is often referred to as a stable and nonamyloidogenic TTR variant that protects TTRV30M carriers from disease [25,48,49,50]. Thus, in order to obtain a PICUP pattern for TTRT119M and compare it with those of TTRwt, TTRV30M, and TTRL55P, we also subjected this nonamyloidogenic variant to acidification at pH 3.6, followed by cross-linking and SDS-PAGE analysis. Figure 5 presents the SDS-PAGE gels for TTRT119M samples incubated at low pH for 12 and 20 days, followed by PICUP. Cross-linked samples are shown in Lane 2 (+). The mobility of the molecular weight markers is shown on the left. No oligomers were formed even after 20 days of incubation.

Given the high stability of TTRT119M tetramers [28,30], extended incubation periods had to be applied (longer than 15 h) at low pH. The results show that only two main gel bands were identified, which were attributed to 1-*mers* and 2-*mers*, with no evidence of other low- or higher-order oligomers. The gel pattern shows that the reaction mixture contains mainly unreacted TTRT119M monomer. This further validates the applicability of the PICUP method and demonstrates the low tendency for aggregation of the TTRT119M variant, which is also correlated with its nonamyloidogenic potential [25,48,49,50]. Additionally, the ThT fluorescence assay (Figure 3) also corroborates these findings since it shows no fluorescence intensity increase or shift of the emission maximum in the presence of the dye, indicating that no amyloid fibril formation occurred.

## 3. Materials and Methods

### 3.1. Materials

Unless otherwise stated, chemicals were purchased from Sigma (St. Louis, MO, USA).

### 3.2. Amyloid Fibril Formation by TTR

Recombinant TTRwt, TTRV30M, TTRL55P, and TTRT119M were produced in an *Escherichia coli* expression system and purified as previously described [28,65]. The protein concentration, as a tetramer, was determined spectrophotometrically at 280 nm using an extinction coefficient of 7.76 × 10^4^ M^−1^ cm^−1^ based on a 55 kDa molecular weight [66]. TTR aggregation was induced by incubation under acidic conditions [26,27,31] for at least 15 h by dilution of a TTR stock in 50 mM glycine buffer containing 0.02% NaN_3_ (pH 3.6) to a final protein concentration of 15 μM for TTRV30M, TTRL55P and TTRT119M, and of 25 μM for TTRwt.

### 3.3. Photo-Induced Cross-Linking of Unmodified Proteins (PICUP)

Photo-induced cross-linking (PICUP) of the aggregating TTR samples was performed as reported by Fancy and Kodadek [32]. In a typical experiment, 15 to 25 μM TTR was cross-linked in the presence of freshly prepared 97 μM Ru(bpy)_3_Cl_2_ (tris(2,2′-bipyridyl)dichlororuthenium(II)) and 2 mM ammonium persulfate. The mixture was irradiated with white light for 1/15 s and quenched immediately in the presence of 580 μM β-mercaptoethanol [32]. Irradiation was performed using a 150 W xenon arc lamp (Oriel) and a camera shutter to control the irradiation time. Light was filtered first through 10 cm of distilled water cooled by an external circulating water bath at 7 °C and then through a 380 nm cutoff filter (Schott & Gen). Samples were positioned in line with the light beam at a distance of 50 cm. Protein controls were prepared under similar conditions and concentrations, with the exception of light exposure. All experiments were performed as independent triplicates and found to be reproducible. To avoid any interference with the oligomerization mechanism, no zwitterionic detergents were added to the mixture, unlike in previous work [51].

### 3.4. SDS-PAGE and Densitometric Analysis

In order to characterize the oligomer distribution in aggregating TTR samples, control samples (non-cross-linked) and cross-linked samples were analyzed by SDS-PAGE. Samples were heated at 95 °C for 10 min in SDS reducing buffer prior to gel loading. Gels were cast with a constant acrylamide concentration of 4% for stacking gels and 5% for running gels and run at a constant voltage of 180 V for 50 min. Gels were stained with silver nitrate [67] and protein bands were analyzed by densitometry using TotalLab TL100 software (Nonlinear Dynamics, Ltd., Newcastle upon Tyne, UK). Densitometric profiles were obtained for each gel lane, and relative intensities were calculated by peak integration after baseline correction.

### 3.5. Thioflavin-T Assay

Concentrated, fresh, and filtered stock solutions of thioflavin-T (ThT) were prepared in 5 mM glycine-NaOH buffer, pH 9.0. ThT concentrations were determined spectrophotometry at 411 nm using an extinction coefficient of 2.2 × 10^4^ M^−1^ cm^−1^ [68]. TTR samples were submitted to final concentrations of 10 μM ThT. The mixture was excited at 450 nm and fluorescence emission was collected between 460 and 560 nm, at room temperature. Fluorescence measurements were carried out in a Varian Cary Eclipse fluorescence spectrophotometer, using excitation and emission slit widths of 5 and 10 nm, respectively.

### 3.6. Transmission Electron Microscopy (TEM)

TTRL55P samples at 15 μM incubated during 5 and 72 h at pH 3.6 and 25 °C were analysed by TEM. TTR samples aliquots of 5 μL were adsorbed onto carbon-coated collodium films supported on 200-mesh copper grids for 1 min. The grids were negatively stained with 1% uranyl acetate and visualized using an EM 902A Zeiss transmission electron microscope operating at 80 kV equipped with a Gatan SC1000 Orius CCD camera.

## 4. Conclusions

To investigate the early stages of the oligomerization mechanism of the amyloidogenic protein transthyretin (TTR) and some of its natural variants (TTRV30M, TTRL55P, and TTRT119M), protein samples were subjected to aggregation-inducing conditions (low pH) at 25 °C prior to PICUP (photo-induced cross-linking of unmodified proteins) analysis. Cross-linking by PICUP enabled the identification of discrete oligomers in solution, revealing the transient molecular species formed during the initial steps of TTR oligomerization. TTRwt, TTRV30M, and TTRL55P presented the same *(n* + 1)*mer* oligomerization mechanism, characterized by a “ladder” of oligomers from 1-*mer* to 8-*mer* (14 to 110 kDa), suggesting a nucleated-independent downhill polymerization, which is consistent with previous work for an engineered monomeric TTR variant (M-TTR) [55] and for TTRwt [26,27]. This successive addition of monomeric subunits (1-*mers*) to the growing LMW (low-molecular-weight) oligomer to at least the formation of an octameric intermediate indicates that the monomer is the building block of LMW TTR oligomers before forming or self-assembling into larger entities. Moreover, in the case of the more amyloidogenic variants TTRV30M and TTRL55P, significant amounts of high-molecular-weight (HMW) aggregates were also observed at the top of the gels, in agreement with the well-known amyloidogenic behavior of these proteins [14,15,16]. Conversely, TTRT119M exhibited the presence of neither HMW nor LMW species other than 1-*mers* and 2-*mers*, even when incubated at low pH for several days, which is also in agreement with its nonamyloidogenic behavior [25,28,48,49,50]. 

TTR amyloid formation has been reported in the literature as consisting of the assembly of TTR monomers [30], dimers [69], or even tetramers [70]. Nevertheless, in our experiments, we observe that, for amyloidogenic variants, initial TTR oligomerization occurs via self-assembly of monomeric units at least up to octamers, prior to the formation of HMW aggregates, which occurs faster for TTRV30M and TTRL55P than for TTRwt, in agreement with the natural amyloidogenicity of these variants. Presently, therapeutic strategies and clinically approved drugs against TTR amyloidosis aim to stabilize the TTR tetramer [71,72] or suppress TTR production [73,74]. Nonetheless, other molecular species, such as monomers or LMW oligomers, can be seen as relevant therapeutic targets and may represent a viable alternative for drug development towards the inhibition of amyloid formation by TTR.

## Supplementary Materials

The following are available online, Figure S1: Effect of irradiation time in the PICUP experiment of TTRV30M and TTRL55P at pH 7.4.
