# Supplementary material for: Oligomerization Profile of Human Transthyretin Variants with Distinct Amyloidogenicity
Source: Molecules. 2020 Dec 3;25(23):5698. doi: 10.3390/molecules25235698 (PMC7730986; doi:10.3390/molecules25235698)
Supplement: Supplementary file 1 [file molecules-25-05698-s001.zip › supp material_v2_track changes.docx]

Oligomerization profile of human transthyretin variants

with distinct amyloidogenicity

Supplementary Materials

**
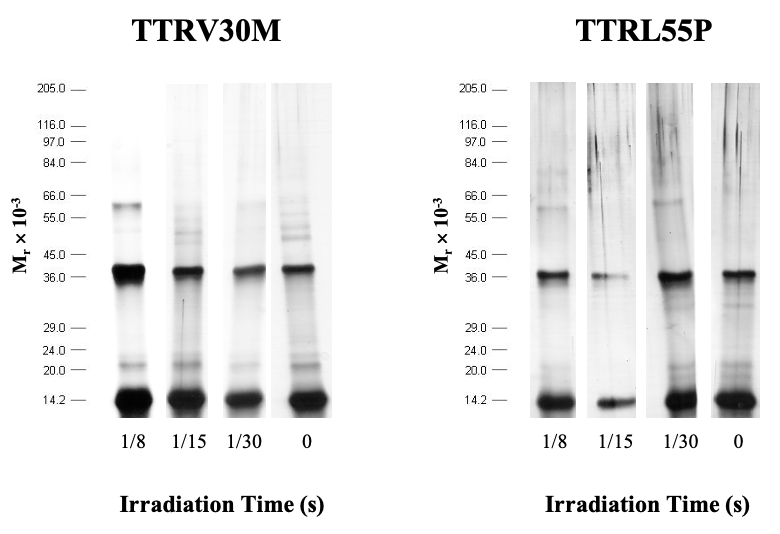
**

**Figure S1.** Effect of irradiation time in the PICUP experiment of TTRV30M and TTRL55P at pH 7.4. TTR samples at 15 μM were incubated for 72 hours, at 25°C. SDS-PAGE of PICUP (photo-induced cross-linking of unmodified proteins) reaction mixtures performed with irradiation times between 1/30 and 1/8 s (7.5% polyacrylamide running gels). A scale of molecular mass markers is shown on the left. The result for a control experiment, in the absence of light irradiation, is shown on the right lane (0 s).
